# Indicators of Healthy Architecture—a Systematic Literature Review

**DOI:** 10.1007/s11524-020-00469-z

**Published:** 2020-09-04

**Authors:** Louis Rice, Mark Drane

**Affiliations:** University of the West of England Bristol, Bristol, UK

**Keywords:** Public health, Architecture, Urban, Wellbeing, Indicators

## Abstract

The design of the built environment plays an important role as a determinant of health. As a society, we are spending an increasing proportion of our time indoors and now spend over 80% of our life inside, so the design of buildings can greatly impact on human health. Accordingly, architecture health indices (AHIs) are used to evidence the effects on human health associated with the design of buildings. AHIs provide quantitative and empirical data upon which architects, clients, users and other stakeholders might monitor and evaluate the healthiness (or otherwise) of architectural design. A systematic literature review was conducted to reveal the current state of knowledge, reveal gaps, explore potential usage and highlight best practice in this area. Whilst there are a number of different health indicators for the built/urban environments more generally, the scope of this review is limited to the scale of a building and specifically those aspects within the remit of a professional architect. In order to examine the range and characteristics of AHIs currently in use, this review explored three electronic bibliographic databases from January 2008 to January 2019. A two-stage selection was undertaken and screening against eligibility criteria checklist carried out. From 15 included studies, 127 documents were identified, and these included 101 AHI. A sample of the most commonly used AHIs was then analysed at an item level. The review reveals that most AHIs are limited to measuring communicable diseases that directly affect physical health through e.g. air quality or water quality. There are very few indicators focusing on factors affecting mental and social health; given the increase in mental and social health problems, greater focus on AHIs related to these health issues should be included. Furthermore, the research reveals an absence of AHIs that address non-communicable diseases (NCDs). As the majority of all poor health outcomes globally are now related to NCDs, and many are associated with the design of the built environment, there is an urgent need to address this situation.

## Overview: The Indoorisation of Society and Ill-Health

The prevalence of diseases and ill-health related to the design of built environment is increasing. Within a social-ecologic systems understanding of public health [1, 2], there is growing concern and awareness of the need to address health challenges related to urban lifestyles [3–5]. The lifestyles of urban populations are particularly germane because the world is rapidly urbanising and over half of the global population now live in cities [6]. By 2050, an additional 2.5 billion people will inhabit urban areas [6]. This societal shift towards cities is relevant because urban populations tend to spend more time indoors than their rural equivalent [7]. Furthermore, even among existing urban populations, the proportion of time spent indoors is increasing [8, 9]. The “indoorisation” of society is a worrying trend for human health for three reasons; firstly there is evidence associating time spent outdoors with improved outcomes for health and wellbeing; and secondly, time spent indoors is negatively associated with a number of health issues [10]. Time spent outdoors, particularly in green and blue spaces, is associated positively for physical, mental and social health [11, 12]. One of the principal hypotheses for this positive relationship is biophilia; the theory being that as humans evolved in external natural settings, humans have an affiliation with other forms of life that promote our own wellbeing [13]. Conversely, spending significant amounts of time indoors can have negative associations for health, particularly for poorer and disadvantaged subpopulations [14–17]. In terms of physical health, air pollution and/or contaminated air are the most significant risks in the short term for respiratory disease, allergy and asthma symptoms [18]; longer term, indoor lifestyles are associated with sedentary lifestyles which contribute indirectly towards other health issues such as obesity, cancer, diabetes which cause mortality and/or cardiovascular disease (CVD) [19–21]. Improving indoor housing quality standards can reduce hospital admissions and thereby reduce pressure on healthcare systems [22]. Indoor environments can impact negatively on poor mental health; key indicators include low levels of daylight, poor quality housing, overcrowding and low levels of agency to control one’s environment [23–25]. Thirdly, the indoorisation of the human population has negatively impacted levels of sociability; we spend less time with others and/or as part of a community, contributing to higher levels of loneliness, isolation and poor social health [26–28]. Whilst there are some policy shifts to encourage society to spend more time outdoors, there is also a clear and urgent need to improve the healthiness of indoor-built environments.

### Architecture and Health

The architecture profession has an important part to play in the puzzle to improve the healthiness of buildings. The Royal Institute of British Architects (RIBA) Code of Professional Conduct includes a public interest principle to protect the wellbeing of the general public and gives priority to this over all other principles [29]. Whilst there are many other stakeholders involved, including engineers, accountants, clients, users, consultants, town planners as well as statutory legislation and other building regulations, it is the architecture profession which invariably has the key role integrating the inputs from this network into a comprehensive and buildable reality. There are a number of challenges facing the implementation of healthier buildings by the architecture profession. First is a lack of knowledge; much more research is required on the complex inter-relationships between built environment factors and their impact on human health. Whilst there are already many existing isolated studies on individual health indicators, more needs to be known about them in combination. Secondly, the architecture profession is not currently required to learn about human health as part of its mandated educational curriculum. This might seem surprising, but at present, none of the professional institutions of architecture specifically requires a knowledge of health and/or wellbeing [30]. This extends to a lack of understanding of population-level approaches and working to address health inequalities which by comparison are integral to public health practice [31]. A paradigm shift in the profession is required in order to address this aspect. Thirdly, the rise of non-communicable diseases is associated with contemporary “lifestyles”; these are highly complex inter-relationships that combine aspects of the built environment with socio-cultural factors, legislation, behavioural patterns, advertising, social media and economics. The drivers of non-communicable diseases are inbuilt into the design DNA of many contemporary cities [32]. Lastly, there are often financial limitations that impinge on the inclusion of healthy design features as “too expensive or costly”. The financial costs of ill-health are already known, for example days lost due to absenteeism at work or the cost to health services treating these illnesses and run into the billions of pounds/dollars/euros [33]. However, these costs have not yet found their way upstream; poor design decisions are made with the full knowledge that they may cause health problems later on, but there is no mechanism available yet to enable greater costs to be allocated upstream. What is needed is an alternative model of economics that better accounts for the cost of ill-health as a result of unhealthy buildings.

### Architecture Health Indicators

The architecture sector has now developed some indictors and indices/tools for measuring, monitoring and evaluating the healthiness of a building. Indicators are increasingly used to measure architectural issues such as sustainability, with a range of individual indicator measurements amalgamated into composite indices or tools [34]. “An indicator is something that provides useful information about a physical, social, or economic system, usually in numerical terms” [35]; it is notable that the terms “tool/indices” are used interchangeably in much of the literature and refer to a composite set of indicators to measure a complex phenomenon. These composite indices are “used normatively, insofar as they are selected to fulfil the purpose of policy and, more generally, decision-making” [36]. This review focuses on composite indices for architectural health rather than individual indicators as this is the approach adopted by the architecture profession and reflects the complex multi-factor problem of health challenges. Accordingly, the World Health Organization defines health as: “a state of complete physical, mental and social well-being and not merely the absence of disease or infirmity” [37]. This definition includes a range of different aspects of health, which, in turn, requires a number of different indicators to capture the complex state of health and wellbeing. There are many “sustainable building indices” developed and are in widespread use throughout the architecture industry. However, there are relatively few indices/tools developed specifically for measuring healthiness of buildings; indeed, many of the available AHIs are based on, or extensions from, sustainable building indicator tools. For this reason (as the article reveals later on), sustainable (aka green or eco) building indicator tools are included in the search criteria for the review.

## Methodology

This review critically explores the current state of architecture health indices. The first objective of the research was to conduct a review of the literature systematically to identify AHI (Table 1). To support this objective, a protocol was developed using Preferred Reporting Items for Systematic Review and Meta-Analysis Protocols (PRISMA-P) guidance [38]. PRISMA-P is a widely used protocol for systematic reviews with an associated methodological and analytical approach established in advance of conducting the review to aid consistency in systematic review methods in health disciplines [ibid]. The objective of this study is to identify AHIs that aim to enable the assessment and evaluation of the impact of architectural design on occupant health. The approach took three stages: first meta-analyses and literature review studies of AHIs were identified for inclusion. Secondly, AHIs were extracted from included studies. Thirdly, item-level criteria were extracted from a sub-set of the most frequently used AHIs globally.

**Table 1:** Summary of included studies (*n* = 15)

Reference	Study design (sourced from)	Aim	Potential AHI reviewed	Named AHI reviewed	Other potential AHIs referenced
Hamid et al. (2014)	Comparative review (database searches)	Review of existing green building rating tools in Malaysia	*n* = 8	Green Globe LEED Green Star NABERS Malaysia specific: GreenRE Green PASS PH JKR GBI	BREEAM HQE CASBEE
Li et al. (2017)	Systematic review (database searches)	Systematic review of comparative analyses of green building assessment methods	*n* = 12	BREEAM, UK LEED, USA CASBEE, Japan Green Star, Australia BEAM Plus, Hong Kong Green Mark, Singapore EcoProfile, Norway DGNB, Germany ESGB or GBL, China SB Tool, International EcoEffect ESCALE	-
Suzer (2015)	Comparative analysis (as part of a wider study) (database searches)	To investigate problems arising from the weighting of sustainability concerns with in LEED	*n* = 5	LEED BREEAM SB Tool CASBEE Green Star	-
Shari and Soebarto (2017)	Literature review (as part of a wider study) (database searches)	Development of sustainability building performance assessment framework for offices in Malaysia	*n* = 6	BREEAM LEED Green Star Green Mark CASBEE GBI	-
Shari and Soebarto (2015)	Comparative review (reference hand search)	Investigate effectiveness of existing building performance assessment systems (BPAS) and their appropriateness for use in Malaysia	*n* = 9	BREEAM LEED SB Tool Green Star Green Mark LEED-India GBES GBI Greenship	-
Retzlaff (2008)	Content analysis (reference hand search)	Developing a framework to support planners in selecting building assessment systems	*n* = 6	EarthCraft Enterprise Community Partners Communities Criteria Green Globes American Lung Association Health House Program LEED (several variants) NAHB Green Building Guidelines	-
Lee (2013)	Comparative review (reference hand search)	Comprehensive review of five building environmental assessment schemes	*n* = 5	BREEAM LEED CASBEE BEAM Plus ESGB	BEPAC, Canada EMAS, Europe GBBC, Korea SBAT, South Africa Green Building Labelling, Taiwan CHEERS, USA Green Building Program, USA SB Tool, international
Alyami and Rezgui (2012)	Comparative review (reference hand search)	Identify similarities and differences between globally prevalent environmental assessment methods, with a view to identifying essential criteria for new schemes including in Saudi Arabia	*n* = 4	BREEAM LEED SB Tool CASBEE	-
Sev (2011)	Comparative analysis (reference hand search)	Investigating the use of most widely used building environmental assessment (BEA) tools and their effectiveness when taken from country of origin and used in developing countries	*n* = 6	BREEAM CEEQUAL LEED CASBEE Green Star SB Tool	EcoProfile PromisE, Finland Green Mark HK-BEAM CEPAS, Hong Kong Green Star SBAT Environmental Status, Sweden
Haapio and Viitaniemi (2008)	Critical review (reference hand search)	Analysing and categorising existing building environmental assessment tools	*n* = 16	ATHENATM Environmental Impact Estimator Building Environmental Assessment Tool (BEAT) BeCost Building for Environment and Economic Sustainability (BEES) BREEAM EcoEffect Eco-Profile Eco-Quantum Envest 2 Environmental Status Model EQUER ESCALE LEGEP Leadership in Energy and Design (LEED) Programmation et Analyse de Projets d’Ouvrages et d’Opérations Soucieux de l’Environnement (PAPOOSE) TEAM	-
Say and Wood (2008)	Industry report/review (reference hand search)	Investigate similarities and differences between four predominant ranking systems	*n* = 5	Green Star BREEAM CASBEE LEED Green Globes	-
Illankoon et al. (2017)	Comparative analysis (reference hand search)	Evaluate the effectiveness of green building rating tools to assess sustainability in buildings by environmental, economic, and social criteria	*n* = 8	America: LEED Europe: BREEAM Asia Pacific: BEAM Plus (Hong Kong) Green Mark (Singapore) CASBEE (Japan) GBI (Malaysia) IGBC (India) Green Star (Australia)	-
Danish Building Research Institute, GXN and 3XN Architects (2012)	Practice guidance (grey lit search)	Provide a comparative overview of key building certifications, with a focus on geographies where Danish practitioners may work	*n* = 45	Refer full text	-
GRESB (2018)	Certification list (grey lit search)	A list of provisionally validated building certification schemes recognised within the GRESB Real Estate Assessment investment benchmark	*n* = 50	Refer full text	-
World Green Building Council (no date)	Website list (grey lit search)	A list of building certification schemes, only those managed by Green Building Councils worldwide	*n* = 45	Refer full text	

### Search Strategy

A search strategy (reported in Appendix) was developed from inclusion criteria under headings of Population, Intervention, Comparison, and Outcome (PICO). Included populations were building occupants; the interventions of interest were AHI as a proxy measure of exposure to building design; no restriction was placed on control/comparator; and included outcome was simply human health. Included study design at first-stage searches was for systematic reviews, meta-analyses, literature reviews, comparative reviews, and critical comparisons of AHI. Three electronic bibliographic databases were searched: Cochrane Central Register of Controlled Trials, MEDLINE (Ovid interface) and Scopus; hand searches of reference lists of included studies were also made. Given the nature of AHI often being published by commercial organisations, professional bodies and non-governmental organisations, grey literature searches were also undertaken from websites of key organisations, notably Green Building Councils globally, and known published tools. The reviews inclusion criteria were for studies in English conducted from January 2008 to January 2019.

Two reviewers assessed studies for inclusion against eligibility criteria (reported in Appendix 2) which focused on buildings (rather than infrastructure or sub-components of buildings), any health, wellbeing or quality of life criteria including sustainability outcomes. Risk of bias in individual studies was not assessed: the purpose of this review was to identify existing indices in order to identify the indicators and criteria being used to assess health, not to assess the validity of those criteria or the overall effectiveness of the tool. A two-stage selection was undertaken with removal of duplicates and initial screening of title and abstract against eligibility criteria using a checklist. This was completed by a first reviewer and validated by the second reviewer who was blind to the first reviewer’s decisions. This was achieved using the web-based Rayyan tool [39]. Screening was then repeated at full-text eligibility stage. Due to the large number of included AHIs, a sub-set of identified AHIs were identified for further analysis to extract summary information about each index which is presented for a narrative synthesis and to extract detailed items. This data was then analysed, with further PICO screening criteria applied at an item level to identify those related to health outcomes of occupants in buildings. This was completed by the first reviewer and validated by the second reviewer. Within this extraction, outcomes were excluded where they did not reference building occupant health.

## Results

**Fig. 1:**
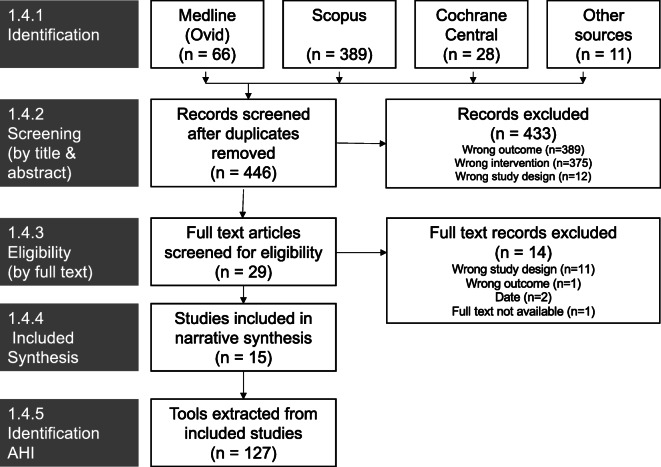
PRISMA flow—included studies

**Fig. 2:**
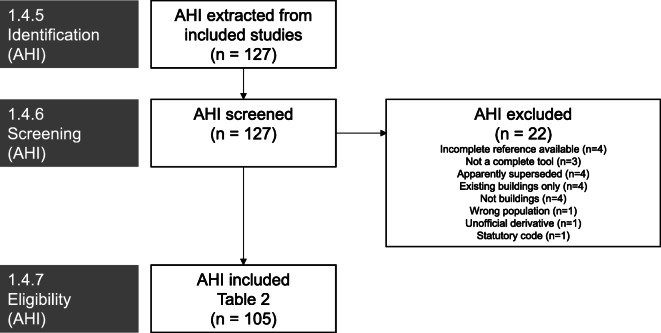
PRISMA flow—included AHIs

Search results are summarised in the PRISMA flow diagram (Fig. 1). Results from databases and other sources including grey literature identified *n* = 446 potentially relevant studies after removal of duplicates. These records were screened by title and abstract by the first reviewer with a representative sample (20%) screened by the second reviewer. The kappa coefficient for this sample was 0.56 between reviewers; all conflicts were subsequently resolved by discussion. Following full-text screening, *n* = 15 sources were included for data extraction. From these sources, *n* = 127 potential AHIs were identified. Following application of screening criteria at the level of AHI, *n* = 105 were included. Screening results are summarised in the PRISMA flow diagram (Fig. 2).

Within the scope of this review, a sub-sample of AHI was purposively sampled for extraction of their entire AHI criteria. Li et al. [40] through a systematic review identified that LEED, BREEAM, CASBEE, Green Star, SB Tool and BEAM Plus were the most frequently studied standards. These indices were selected for criteria extraction along with two more recently developed (but under-reported) AHIs that have a specific focus on health, namely Well Building Standard (WBS) and Fitwel. Through a lens of architectural design practice, these two AHIs are important to practitioners and compared with previous studies, the inclusion of two newer standards focussed on health is novel. In total, *n* = 263 individual criteria across *n* = 8 AHIs related to the health of building occupants were identified. A summary of the number of these and how they are allocated across the tools extracted is included at Table 2.

**Table 2:** Summary of included health criteria within widely used AHI

AHI/concept	No. of included health criteria
LEED	9
Indoor environmental quality	9
BREEAM	4
Health and wellbeing	4
CASBEE	18
LR: environmental load reduction of building	1
Q: environmental quality of building	17
Green Star	19
Emissions	1
Indoor environment quality	17
Land use and ecology	1
BEAM Plus	20
Site aspects (SA)	1
Water use (WU)	1
Indoor environmental quality (IEQ)	18
SB tool	36
D indoor environmental quality	18
E service quality	14
F social, cultural and perceptual aspects	4
Well Building Standard	128
Air	44
Comfort	22
Fitness	10
Light	18
Mind	14
Nourishment	6
Water	14
Fitwel	29
4. Entrances and ground floor	6
5. Stairwells	5
6. Indoor environment	2
7. Workspaces	3
8. Shared spaces	7
9. Water supply	2
10. Food services	1
11. Vending machines and snack bars	1
12. Emergency procedures	2

## Findings and Discussion

The findings of the review have been categorised into four themes that emerged from the data: scale, scope, source and scoring. The findings and discussion explore each of these themes in turn.

### Scale

The reviews in the literature (particularly when hand searches are excluded) as presented in Table 1 reveal that a limited number of AHI systems appear repeatedly in research. Five specific AHIs appeared most frequently in the literature, namely BREEAM, LEED, SB Tool, CASBEE, and Green Star. These emerged in almost all of the searches and reflect that these tend to be industry standards in different regions globally. BREEAM (building research establishment environmental assessment method) was developed by the UK Building Research Establishment (BRE) and grew from an energy-efficiency measurement model into one that includes health indicators. BREEAM is more prevalent in the UK and the EU. LEED (the leadership in energy and environmental design) standard was developed by the US Green Building Council (USGBC) and is widely used in the USA. Both BREEAM and LEED have multiple variants including global regional variants—such variants were excluded from this study. The International Initiative for a Sustainable Built Environment developed the SB Tool. CASBEE (comprehensive assessment system for built environment efficiency) was developed in Japan and is used in Japan and South East Asia. Green Star was created by the Green Building Council Australia and is mostly used in Australasian nations. Fitwel and WBS are more commonly adopted in Northern America. Whilst there are other AHIs that have been developed, they tend not to feature consistently in the literature reviews. These findings reveal that only a handful of AHIs tend to dominate within the construction industry and use by the architectural profession but are restricted to regional (often continental) areas.

### Scope

The AHIs are composites of individual indicators, which, when broken into the individual components, reveal further similitude. Each of the 5 dominant AHIs (BREEAM, LEED, SB Tool, CASBEE and Green Star) tends to identify the same indicators of health. In general (although a different terminology is sometimes used across these systems), 5 subject areas are measured: air quality, lighting levels, acoustics, thermal comfort and safety.

The most prevalent indicator is related to air quality; these come under various guises, including, for example “formaldehyde concentration in air” and “total volatile organic compounds concentration in air”. However, all are related through the concern that buildings are becoming increasingly air-tight (to reduce heat loss) and this might be inadvertently leading to poor air quality and thence to ill-health. The importance of lighting levels is mostly related to ensuring that eyesight is not damaged whilst working from being too bright or too dim. Acoustic indicators try to ensure no damage to hearing occurs from excessive noise or noise pollution. Thermal comfort attempts to control excessive heating or cooling and those temperatures inside are appropriate for the particular functions within that building. Lastly, the thematic section safety aims to reduce injuries and accidents from preventable falls, slips or trips and is often covered in national “Health and Safety” legislation. These 5 thematic areas upon which the AHIs focus are mostly associated with reducing or preventing communicable diseases and/or injuries. However the WHO definition of health requires “a state of complete physical, mental and social well-being and not merely the absence of disease or infirmity”; the findings reveal that the current AHIs mostly focus on absence of disease but contain few or no indicators to capture the fuller range of “complete physical, mental and social well-being”. This is significant because the burden of ill-health globally is now related to non-communicable diseases, rather than communicable diseases. Furthermore, some of the health indicators included in the AHI are not within the remit of an architect or building designer. There are indicators such as “Meal Sizes” (WBS) and “Tobacco Smoke Control” (LEED) that, whilst pertinent to health, are not related to the design of a building. These indicators are more in the control of the eventual users of the building but would not affect the design, layout or fabric of the building. There is currently paucity in what AHIs measure in terms of healthy architecture pertaining to the wider determinants of health and lifestyle diseases. There is a need for joining up the design stages of a building with the operation of the building and post-occupancy healthiness of a building. Furthermore, it is necessary to also “join up” policies for healthy architecture and healthy cities, to facilitate a coherent and consistent approach to the design of built environments. Particularly as society spends so much time indoors, it is necessary to include the design of buildings as part of a broader “health in all policies” and “health in all designs” approach, if we are to successfully combat the complex determinants of lifestyle diseases. In order to reduce the current burden of ill-health that afflicts the majority of the population, there is a need to update and expand AHIs to include more indicators focused on evaluating non-communicable diseases related to the design of the buildings.

### Source

The 5 most commonly used AHI tools were developed from systems intended to measure the level of “sustainability” of a building. These AHIs were initially developed with a focus on energy efficiency of building design, and later widened to include other sustainable features such as carbon footprint and whole life cycle of construction and refurbishment. BREEAM which was developed in 1990 was the first widely used sustainability evaluation tool and was emulated shortly afterwards by LEED and SB Tool; CASBEE and Green Star were launched in 2003 [41]. Many of these AHIs have subsequently added more of a focus on indicators targeted towards measuring and evaluating health and wellbeing. Whilst not reducing the importance of building in an environmentally sustainable manner, it does nonetheless indicate that the AHI scores and evaluations mostly concern factors other than human health and wellbeing, and that health aspects get “lost” amidst all the other non-health issues. It is perhaps appropriate for different scores or evaluations to be performed separately for energy efficiency aspects and health aspects so that a clearer picture of each is presented to, and comprehensible by, clients, architects, users and other stakeholders.

### Scoring

BREEAM scoring is undertaken at the individual indicator level and a composite sum of various categories are weighted to calculate an overall percentage score. Individual projects receive a BREEAM rating: outstanding (≥ 85%), excellent (≥ 70%), very good (≥ 55%), good (≥ 45%), pass (≥ 30%), unclassified (< 30%). In addition to these percentages, there are minimum standards required and the requirement varies by rating. LEED uses a points-based system (individual item points available range from 1 to 18) which are awarded at the level of individual indicators and the total sum gives a general score. A number of items are not scored but are required to be achieved. Individual buildings receive a LEED rating within the following ranges: platinum (80 points), gold (60–79 points), silver (50–59 points), certified (40–49 points). The SB Tool requires scoring undertaken at the individual item level but unlike other tools, the weighting of each item is based on an algorithm that accounts for local effects, extent of effect, duration of effect, intensity of effect and links to key issue areas. The tool is flexible in that it can be modified for different contexts and has minimum standards for certain indicators, and each building is given a numerical rating/score. CASBEE assesses each indicator on a scale of 1–5 with 1 equating to minimum regulatory compliance which is then compiled into a composite overall score. Projects are given one of five grades: excellent, S (BEE ≥ 3), very good, A (BEE 1.5–3.0), good, B+ (BEE 1.0–1.5), fairly poor, B− (BEE 0.5–1.0), poor, C (BEE< 0.5). Green Star projects are scored up to 100 points across all items. Buildings are awarded a rating on a scale of 1–6 stars: 1 star (minimum practice), 2 stars (average practice), 3 stars (good practice), 4 stars (best practice), 5 stars (Australian excellence), 6 stars (world leadership). BEAM Plus scores are undertaken at the individual item level and the sum of each category is weighted to calculate an overall percentage score. Additionally, certain minimum standards are required within each category to achieve these grades and individual projects receive a BEAM Plus grade: platinum/excellent (≥ 75%), gold/very good (≥ 65%), silver/good (≥ 55%), bronze/above average (≥ 40%). Fitwel scores using a rating of 3 stars (Fitwel score 125–144); 2 stars (Fitwel score 105–124); 1 star (Fitwel score 90–104). Well Building Standard assesses each criterion on a scale of 1–10 and the overall composite is graded on a scale of silver, gold, or platinum. All applicable preconditions for the project type must be achieved. For gold, 40% of applicable optimisations must be achieved, and for platinum, 80% of applicable optimisations must be achieved. The certification is valid for 3 years and must be reassessed after this time.

The scoring systems are arguably overly positive in their choice of ratings, particularly the purposive use of language. LEED and BEAM Plus use terms such as “gold” and “silver” which all suggest a degree of excellence, but potentially sound significantly poorer than when termed “second” or “third” rate. Similarly, the scores for BREEAM only require a score of 30% in order to pass, which is a relatively low benchmark for examinations. CASBEE is perhaps the most direct in its labelling of scores, particularly with the use of the term “poor” to describe its lowest category of building. There is also evidently a divergence of scoring systems, as different scores and systems are applied. Furthermore, it is difficult to ascertain whether gold in one AHI would similarly merit gold if applying a different AHI. There is a need for greater clarity of benchmarking and scoring across systems to improve comprehensibility and legibility for users. Arguably many users are now familiar with the “fridge-sticker” type ratings/scores (from A to F/G, and colour coded from green through yellow to red) which are used on many products globally from domestic appliances, buildings and even the healthiness of food. It might be more appropriate if these systems be applied to AHI scores and ratings, as this would be more equitable and comprehensible by building users.

### Future Research

The research highlights for the first time the limited scale and extent of health criteria applied in the field of architectural design. There is currently a very narrow focus on easily quantifiable indicators such as air quality or prevention of injuries. However, there is very little attention with respect to non-communicable diseases, despite the risks these pose to the population more generally. Action on non-communicable diseases requires changes at an architectural scale as part of a holistic and comprehensive public health strategy to improve wellbeing. There are already calls for an expansion of the public health profession to include architects and built environment professionals [42]. Health-*ifying* the architectural profession would result in a paradigm shift in its values and ethics, perhaps requiring a Hippocratic Oath when entering the profession. The call for “health in all policies” necessitates a concomitant “health in all designs” programme to ensure joined-up action across a range of scales including product design, architecture, urban design, town planning and landscape design. There is considerable capacity and urgency for further insight into this area, from academic research and exemplars from architectural practice.

## Conclusion

Architectural health indices (AHIs) are composite measurement tools used to evaluate the healthiness of building design. AHIs are becoming more prevalent in the design of buildings and of use by the architectural profession. Building design can impact on human health in numerous and complex ways. The systematic literature review provides the most comprehensive list of AHIs currently available which should be of value to other researchers as a source for further investigation. The findings highlighted 4 key themes that emerged related to scale, scope, source and scoring. There is a limited scale evident in use, with the majority of all research involving only a handful of AHIs (namely BREEAM, LEED, SB Tool, CASBEE and Green Star) plus the newer Fitwel and WBS schemes. These AHIs are the most widely used in industry and are the focus of most independent scholarly research. It is notable that the source of each of these AHIs emerged from evaluation tools where the main focus was on measuring environmental efficiency, material sustainability and energy performance of buildings. There is a limited degree of scope to most AHIs, even when they contain indicators related to human health; the majority of indicators relate to broader issues of sustainability. Those indicators that are included are targeted towards the measurement of communicable diseases and prevention of accidents. The findings highlight that there is limited accounting of factors related to non-communicable diseases, which restricts the utility and relevance of AHI. The scoring systems vary across schemes, from percentages, descriptors (platinum, gold/excellent, poor etc.), stars to ratios. The divergent scoring approaches to AHI make it difficult to compare between different regions and are difficult for users to understand the relative/absolute merits thereof. As many health issues globally are related, directly or indirectly, to the design of the built environment, there is a need for better and more comprehensive AHI to be developed to allow for fuller evaluation of the health implications of architecture.

## Appendix Search strategy

Population:

Not restricted. Whole population.

Intervention/exposure:

“tool OR benchmark OR indicator OR index OR indicies OR measure OR metric OR profile OR assessment OR score OR standard OR system OR certification”

AND

(building w/3 design) OR (interior w/3 design) OR (spatial w/3 design) OR (architectur* w/3 design) OR (development w/3 design) OR “green building”.

Comparison:

Not restricted. Default: no intervention/exposure.

Outcome:

Not restricted.

Generic* search syntax:

**Table:**

Population	Intervention	Comparison	Outcome
And	And	And	And
[any]	HCMT	[any]	[any]

Generic* search filters:

**Table:**

Study design	“Systematic review” “Meta analysis” “Literature review” “Review of the literature” “Comparative review” “Critical comparison”	Date range	01/01/2008–present**
Language	English	Publication type	Peer reviewed journals

*Specific syntax and filters vary by database. For example, Medline (Ovid interface) only allows full year search

**Searches conducted 25 January 2019
